# Supplementary material for: Diurnal and seasonal molecular rhythms in human neocortex and their relation to Alzheimer's disease
Source: Nat Commun. 2017 Apr 3;8:14931. doi: 10.1038/ncomms14931 (PMC5382268; doi:10.1038/ncomms14931)
Supplement: Supplementary Information — Supplementary Figures. [file ncomms14931-s1.pdf]

SUPPLEMENTARY FIGURES

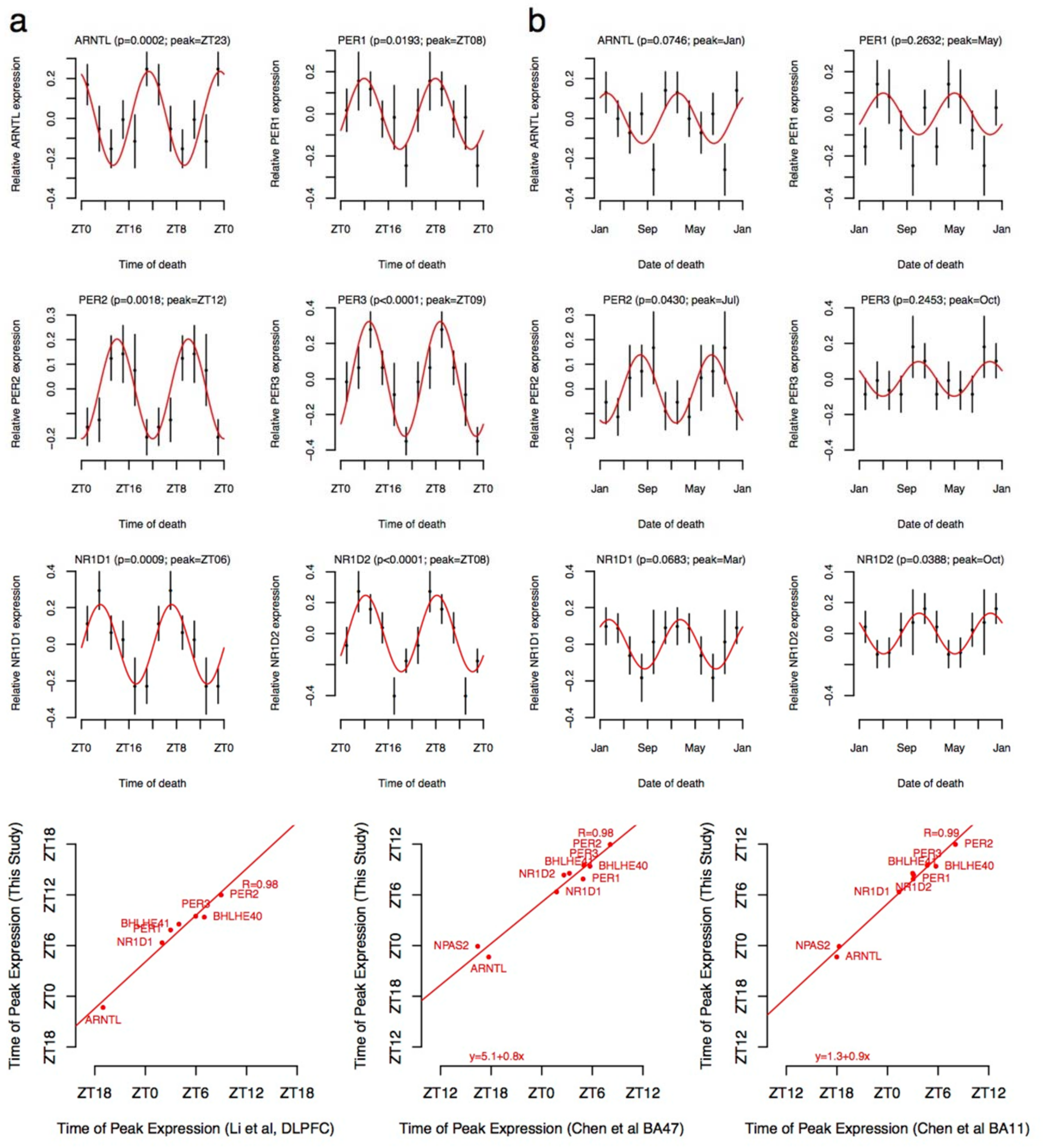

Supplementary Figure 1: Diurnal and seasonal rhythms of clock gene expression. Diurnal rhythms referenced to midnight local clock time. Same as Fig. 3, but reanalyzed with ZT0 = midnight, local clock time. **(**a-b): Relative expression by time of death for several genes known to be involved in the regulation of the mammalian circadian clock (a) and month of death (b) for the several genes known to be involved in the regulation of the mammalian circadian clock. Data plotted in 4-hour (a) or 2-month (b) bins. Dots indicate means and bars indicate standard errors of the mean. Data are double plotted. Red lines indicate best-fit cosine curve. P-values for diurnal (a) or seasonal (b) rhythmicity are as calculated as described in the text using a model considering diurnal and seasonal rhythmicity concurrently, and adjusted for demographic and methodological covariates. (c): Correlation between the peak expression times of known circadian clock genes in our dataset (y-axis) and published human prefrontal cortex datasets (x-axis). Red line indicates best-fit linear regression.

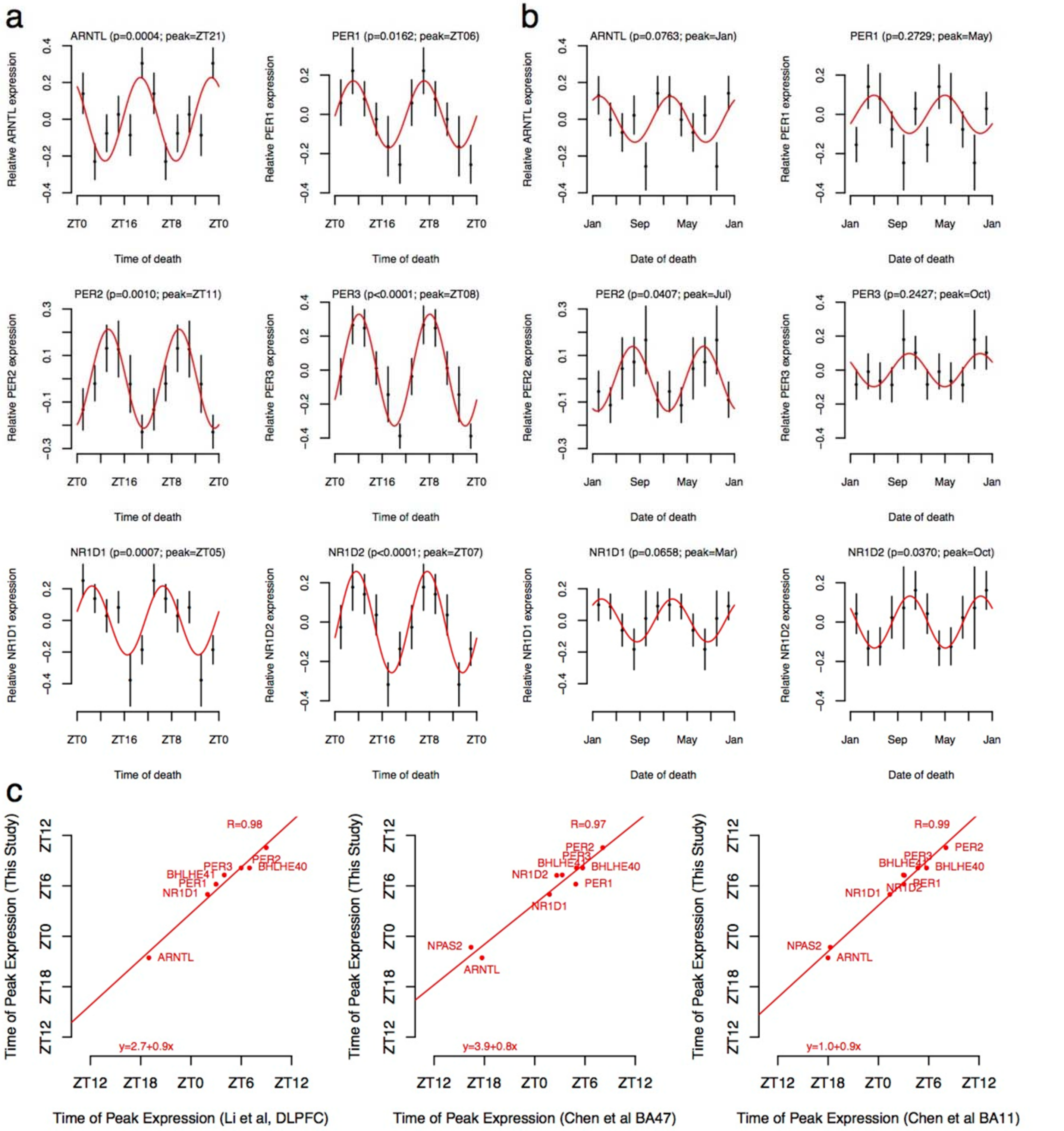

Supplementary Figure 2: Diurnal and seasonal rhythms of clock gene expression. Diurnal rhythms referenced to the midpoint of the dark period. Same as Fig. 3, but reanalyzed with ZT0 = the midpoint of the dark period. **(**a-b): Relative expression by time of death (a) and month of death (b) for several genes known to be involved in the regulation of the mammalian circadian clock. Data plotted in 4-hour (a) or 2-month (b) bins. Dots indicate means and bars indicate standard errors of the mean. Data are double plotted. Red lines indicate best-fit cosine curve. P-values for diurnal (a) or seasonal (b) rhythmicity are as calculated as described in the text using a model considering diurnal and seasonal rhythmicity concurrently, and adjusted for demographic and methodological covariates. (c): Correlation between the peak expression times of known circadian clock genes in our dataset (y-axis) and published human prefrontal cortex datasets (x-axis). Red line indicates best-fit linear regression.

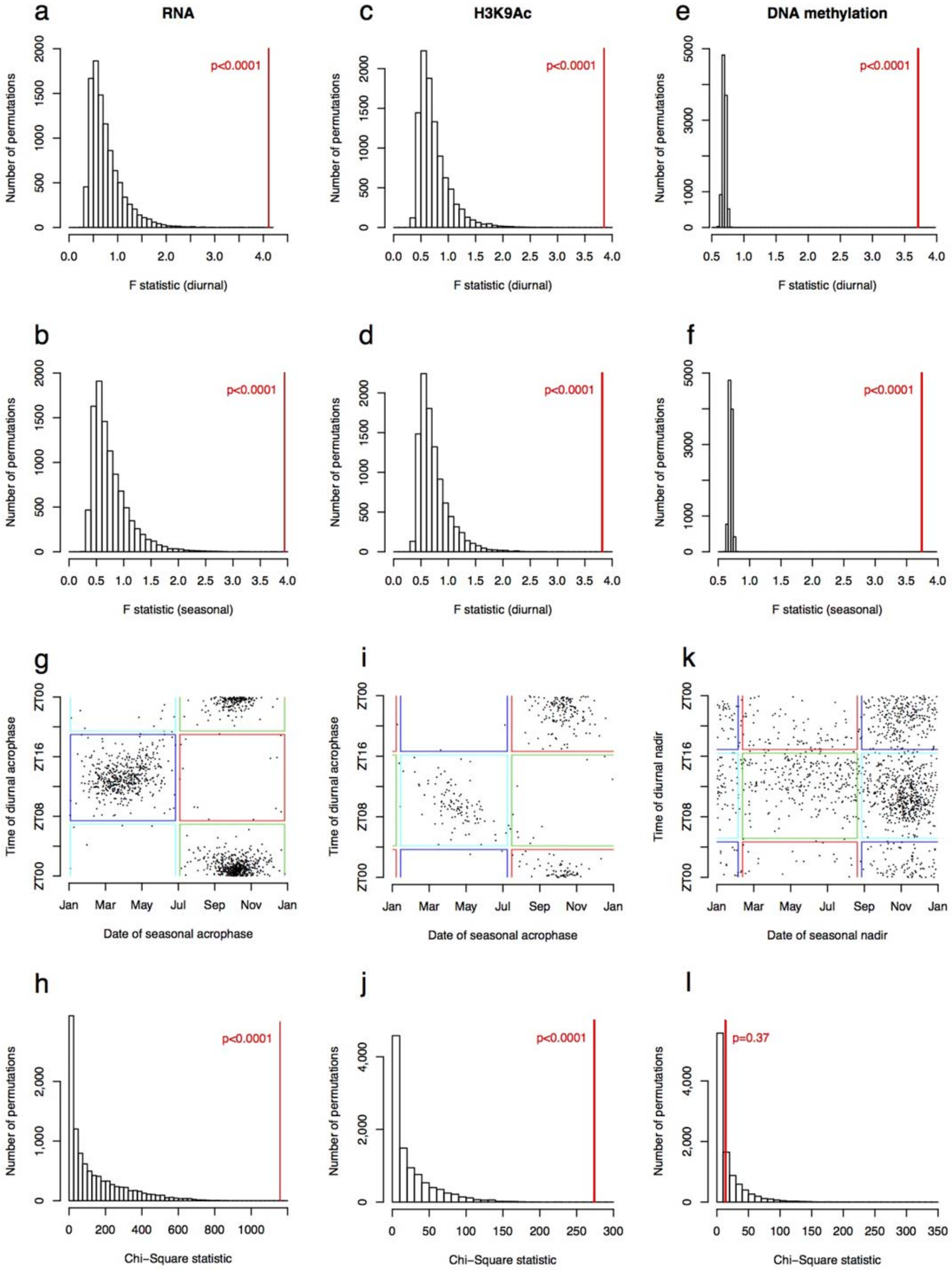

Supplementary Figure 3: Diurnal and seasonal rhythmicity in the transcriptome and epigenome considering only the most rhythmic sites. (a): Observed (red) vs. expected (black) median F-statistic for diurnal rhythmicity considering all transcripts with $p<0.05$ by the F-test. Null distribution estimated by consideration of 10,000 empiric null datasets generated by randomly shuffling times of death. (b): as in (a) but for seasonal rhythms. (c-d): as in (a-b) but for H3K9Ac peaks. (e-f): as in (a-b) but for DNA methylation sites. (g): Association between time of diurnal vs. seasonal acrophases. Each dot represents a single transcript. Colored boxes depict empirically derived clusters. (h): Observed (red line) vs. expected (black bars) Chi-square statistic for association between timing of diurnal and seasonal rhythms. Expected distribution empirically derived from 10,000 permuted null datasets generated by randomly shuffling times and dates of death. (i-j): same as (g-h) but for H3K9Ac peaks. (k-l): same as for (g-h) but for the diurnal and seasonal nadirs of individual DNA methylation sites.

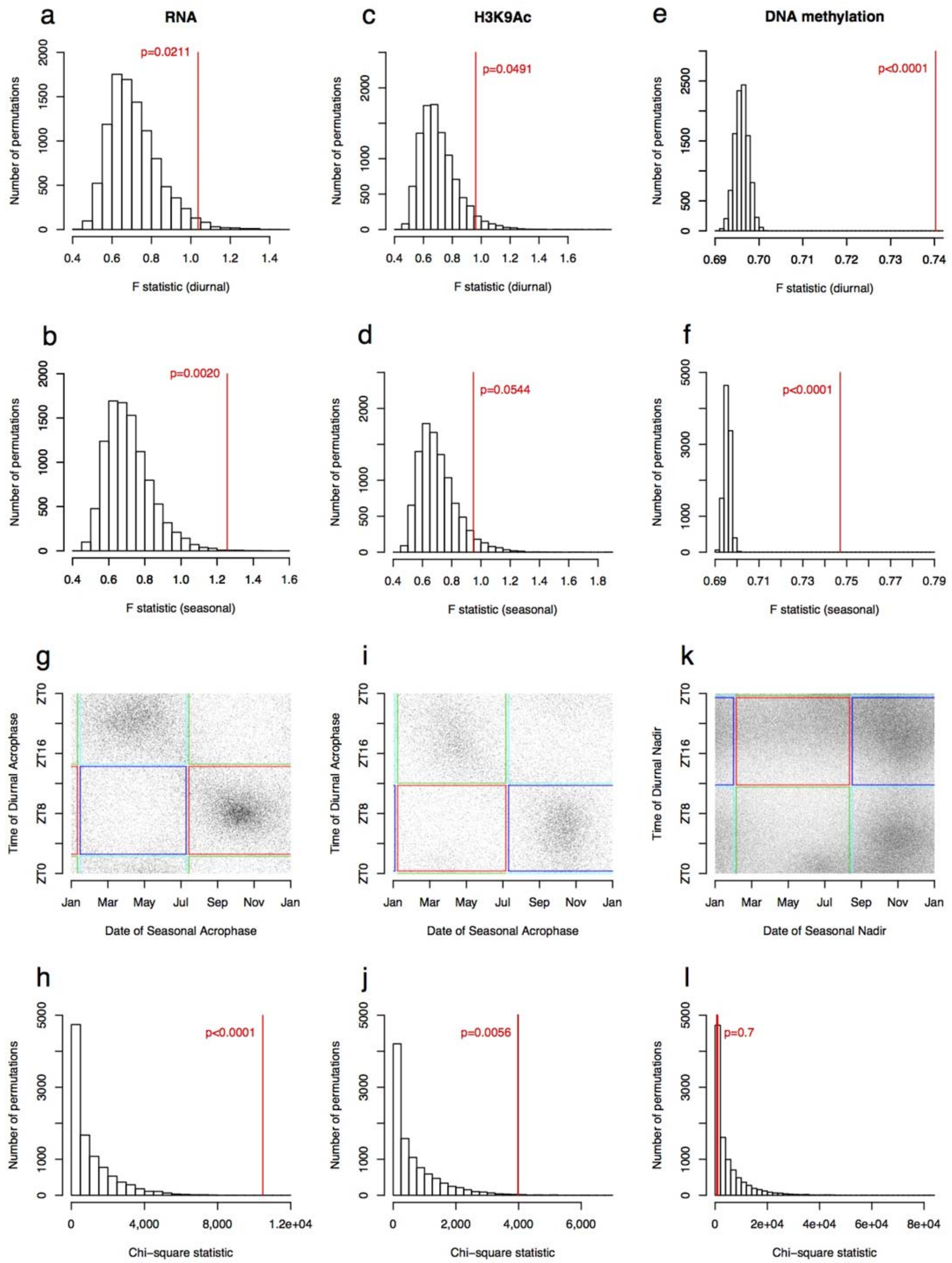

Supplementary Figure 4: Diurnal and seasonal rhythmicity in the transcriptome and epigenome with diurnal rhythms referenced to local clock time. Same as Fig. 4, but reanalyzed with ZT0 = midnight, local clock time. **(**a): Observed (red) vs. expected (black) median F-statistic for diurnal rhythmicity considering all transcripts together. Null distribution estimated by consideration of 10,000 empiric null datasets generated by randomly shuffling times of death. (b): as in a but for seasonal rhythms. (c-d): as in (a-b) but for H3K9Ac peaks. (e-f): as in (a-b) but for DNA methylation sites. (g): Association between time of diurnal vs. seasonal acrophases. Each dot represents a single transcript. Colored boxes depict empirically derived clusters. (h): Observed (red line) vs. expected (black bars) χ^2 statistic for association between timing of diurnal and seasonal rhythms. Expected distribution empirically derived from 10,000 permuted null datasets generated by randomly shuffling times and dates of death. (i-j): same as (g-h) but for H3K9Ac peaks. (k-l): same as for (g-h) but for the diurnal and seasonal nadirs of individual DNA methylation sites.

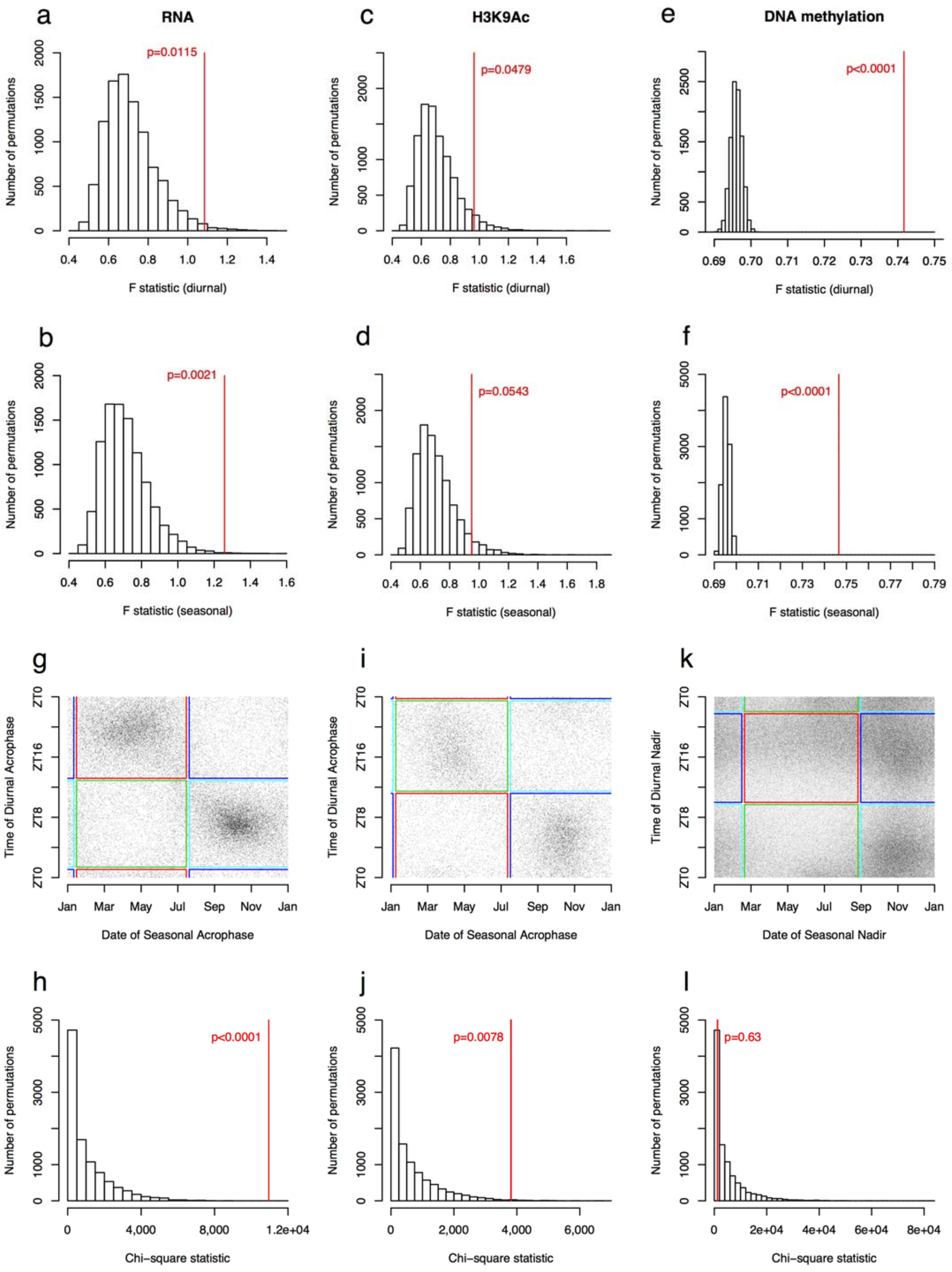

Supplementary Figure 5: Diurnal and seasonal rhythmicity in the transcriptome and epigenome with diurnal rhythms referenced to mid point of dark period. Same as Fig. 4, but reanalyzed with ZT0 = midpoint of dark period. **(**a): Observed (red) vs. expected (black) median F-statistic for diurnal rhythmicity considering all transcripts together. Null distribution estimated by consideration of 10,000 empiric null datasets generated by randomly shuffling times of death. (b): as in a but for seasonal rhythms. (c-d): as in (a-b) but for H3K9Ac peaks. (e-f): as in (a-b) but for DNA methylation sites. (g): Association between time of diurnal vs. seasonal acrophases. Each dot represents a single transcript. Colored boxes depict empirically derived clusters. (h): Observed (red line) vs. expected (black bars) χ^2 statistic for association between timing of diurnal and seasonal rhythms. Expected distribution empirically derived from 10,000 permuted null datasets generated by randomly shuffling times and dates of death. (i-j): same as (g-h) but for H3K9Ac peaks. (k-l): same as for (g-h) but for the diurnal and seasonal nadirs of individual DNA methylation sites.

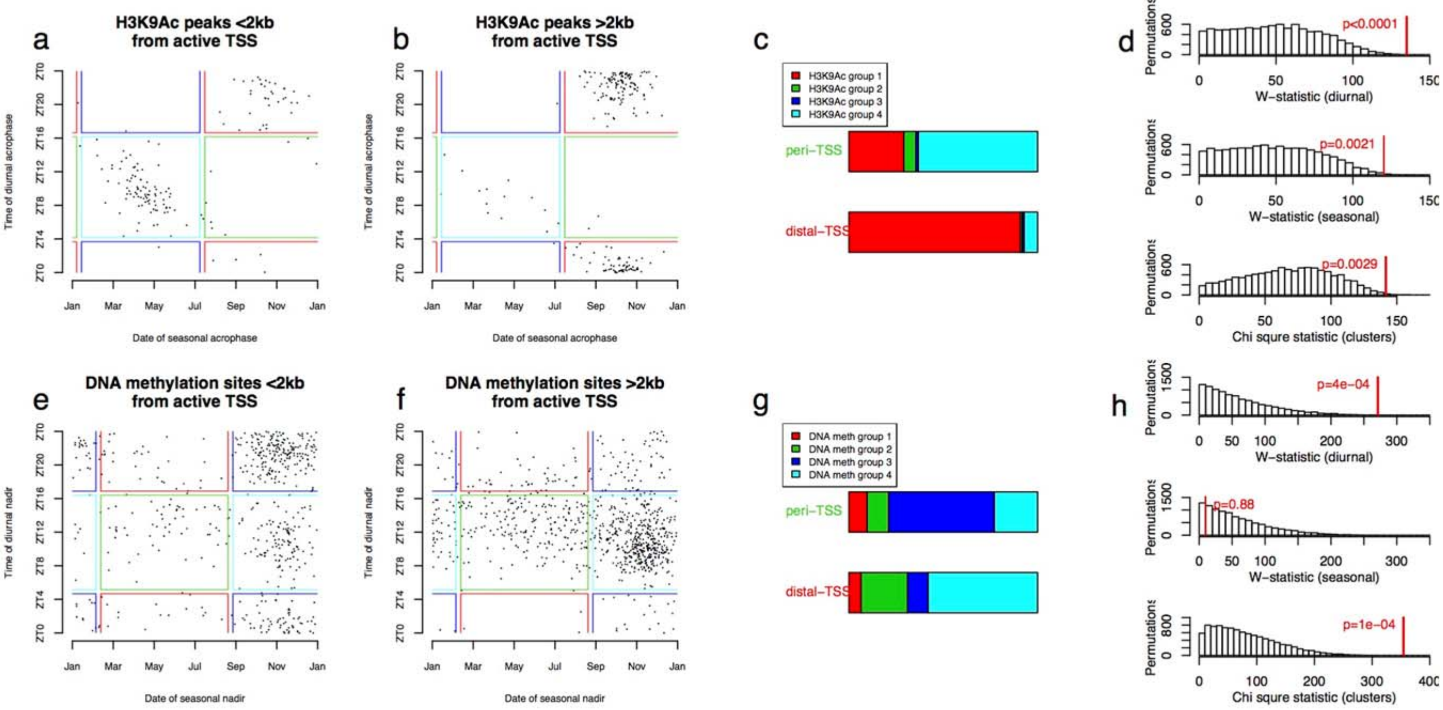

Supplementary Figure 6: Physical position and diurnal and seasonal rhythms in the epigenome considering only the most rhythmic sites. Same a Fig. 5 but considering only those sites with $p<0.05$ for diurnal and seasonal rhythmicity by the F-test. (a): Association between time of diurnal vs. seasonal acrophase of H3K9Ac peaks less than 2kb from active transcription start sites. Each dot represents a single H3K9Ac peak or DNA methylation site. Colored boxes depict empirically derived clusters. (b): same but for H3K9Ac peaks >2kb from active transcription start sites. (c): Temporal classification of H3K9Ac peaks less than or more than 2kb from active transcription start sites. (d): Observed (red line) vs. expected distribution of W-statistic for angular distribution of diurnal acrophases, W-statistic for angular distribution of seasonal acrophases, and Chi-square statistic for temporal classification of H3K9Ac acrophases comparing sites less than or more than 2kb of active transcription start sites. (e-h): same as for (a-d) but for DNA methylation sites.

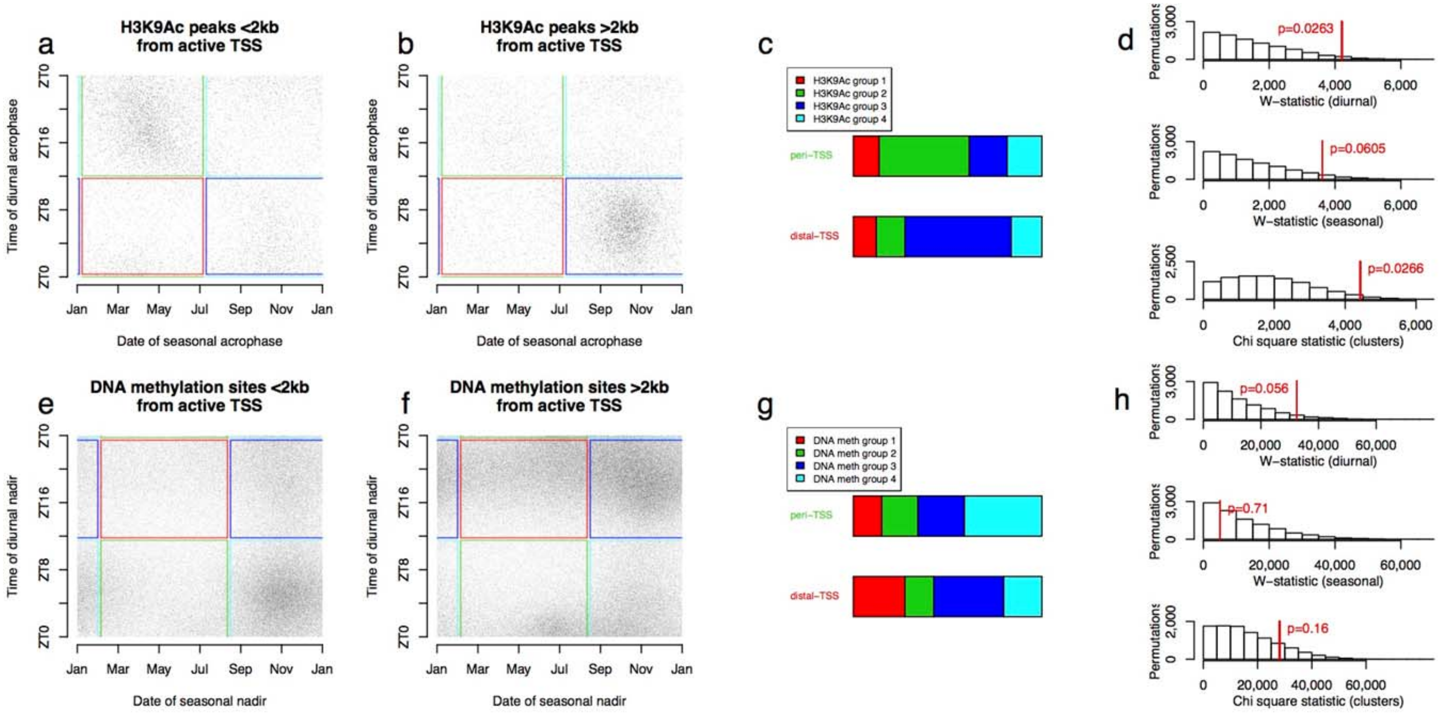

Supplementary Figure 7: Physical position and diurnal and seasonal rhythms in the epigenome with diurnal rhythms referenced to local clock time. Same as Fig. 5, but reanalyzed with ZT0 = midnight, local clock time. (a): Association between time of diurnal vs. seasonal acrophase of H3K9Ac peaks less than 2kb from active transcription start sites. Each dot represents a single H3K9Ac peak. Colored boxes depict empirically derived clusters. (b): same but for H3K9Ac peaks >2kb from active transcription start sites. (c): Temporal classification of H3K9Ac peaks less than or more than 2kb from active transcription start sites. (d): Observed (red line) vs. expected distribution of W-statistic for angular distribution of diurnal acrophases, W-statistic for angular distribution of seasonal acrophases, and Chi-square statistic for temporal classification of H3K9Ac acrophases comparing sites less than or more than 2kb of active transcription start sites. (e-h): same as for (a-d) but for DNA methylation sites.

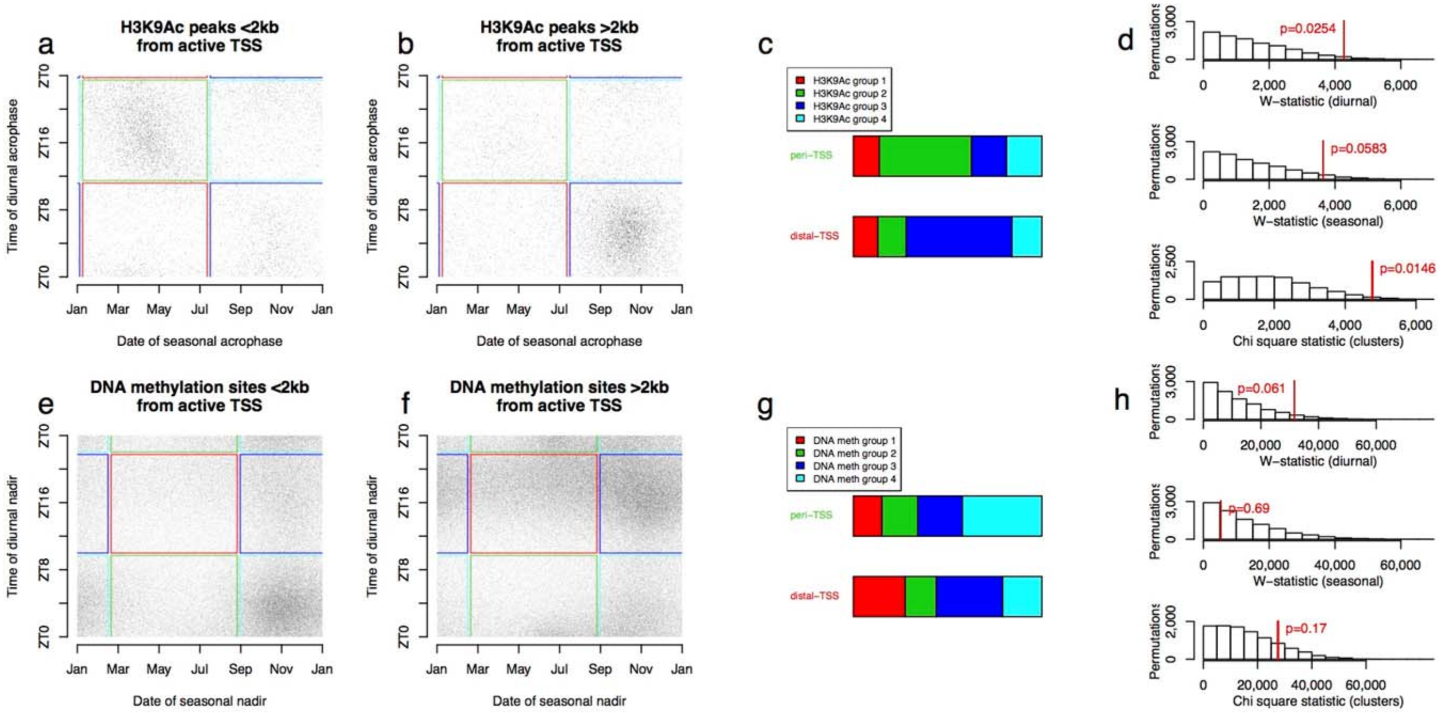

Supplementary Figure 8: Physical position and diurnal and seasonal rhythms in the epigenome with diurnal rhythms referenced to the midpoint of the dark period. Same as Fig. 5, but reanalyzed with ZT0 = midpoint of the dark period. (a): Association between time of diurnal vs. seasonal acrophase of H3K9Ac peaks less than 2kb from active transcription start sites. Each dot represents a single H3K9Ac peak. Colored boxes depict empirically derived clusters. (b): same but for H3K9Ac peaks >2kb from active transcription start sites. (c): Temporal classification of H3K9Ac peaks less than or more than 2kb from active transcription start sites. (d): Observed (red line) vs. expected distribution of W-statistic for angular distribution of diurnal acrophases, W-statistic for angular distribution of seasonal acrophases, and Chi-square statistic for temporal classification of H3K9Ac acrophases comparing sites less than or more than 2kb of active transcription start sites. (e-h): same as for (a-d) but for DNA methylation sites.

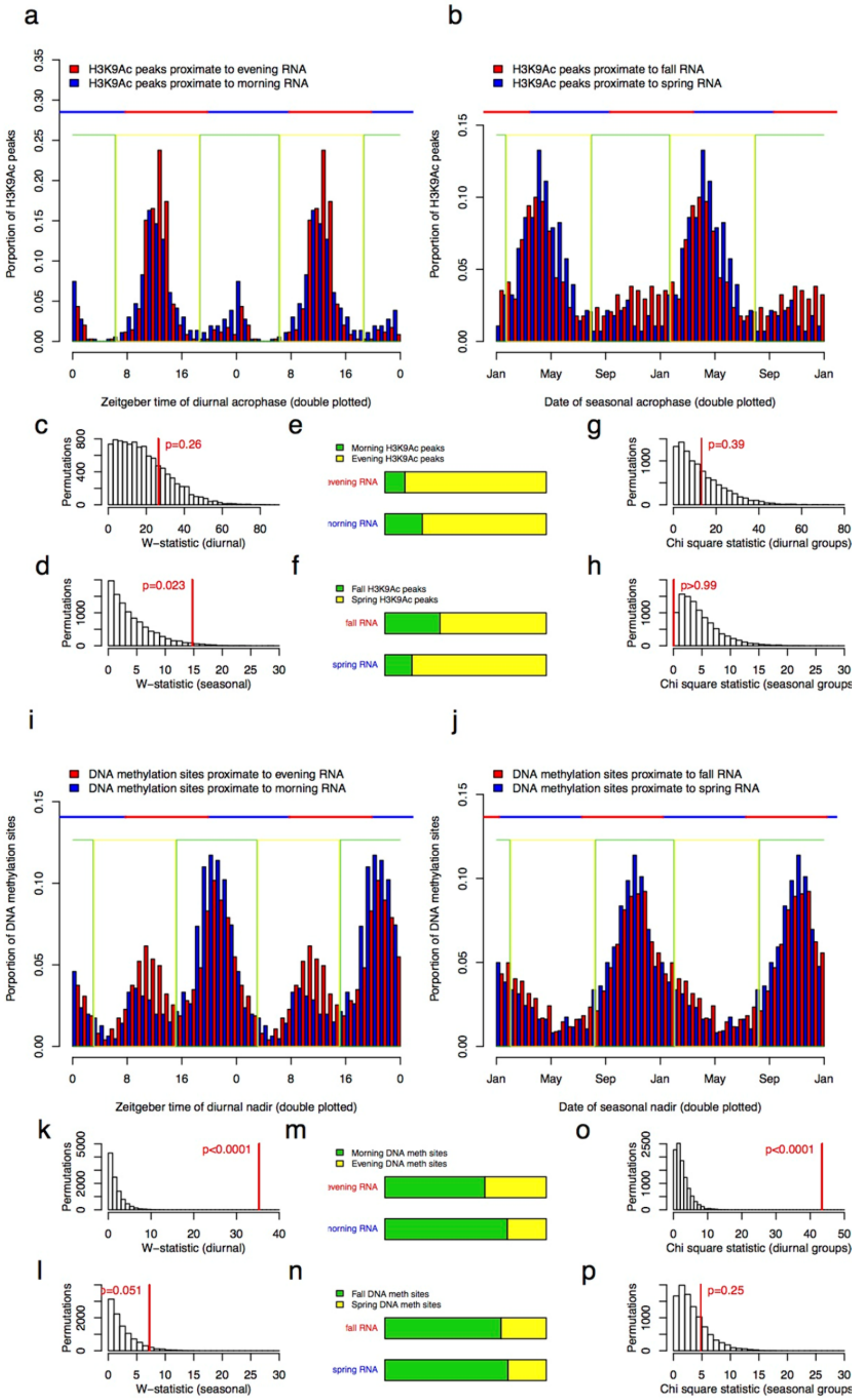

Supplementary Figure 9: Association between rhythms in the transcriptome and epigenome considering only the most rhythmic sites. Same as Fig. 6, but considering only those sites with $p<0.05$ for diurnal or seasonal rhythmicity by the F-test. (a): temporal distribution of H3K9Ac diurnal acrophases for peaks within 2kb of the transcription start sites of evening peaking (red) and morning peaking (blue) transcripts. Data are double plotted. Horizontal line indicates temporal boundaries of the associated transcript classes. Green and yellow boxes indicate temporal boundaries of H3K9Ac classes (evening yellow, morning green). (b): same as (a) but for seasonal acrophases. (c): observed vs. expected W-statistic comparing the diurnal distributions of H3K9Ac acrophases for peaks within 2kb of the transcription start sites of evening peaking and morning peaking transcripts. Expected distribution derived from 10,000 permuted null datasets generated by randomly shuffling times of death. (d): same as (c) but for seasonal H3K9Ac acrophases. (e): diurnal temporal classification of H3K9Ac peaks near evening vs. morning peaking transcripts. (f): same as (e) for seasonal temporal classification. (g): observed vs. expected Chi-square statistic comparing the diurnal temporal classification of H3K9Ac peaks near evening vs. morning peaking transcripts. (h): same as (g) but for seasonal temporal classification. (i-p): same as (a-h) but for the diurnal and seasonal nadirs of DNA methylation sites near evening vs. morning, or fall vs. spring peaking transcripts.

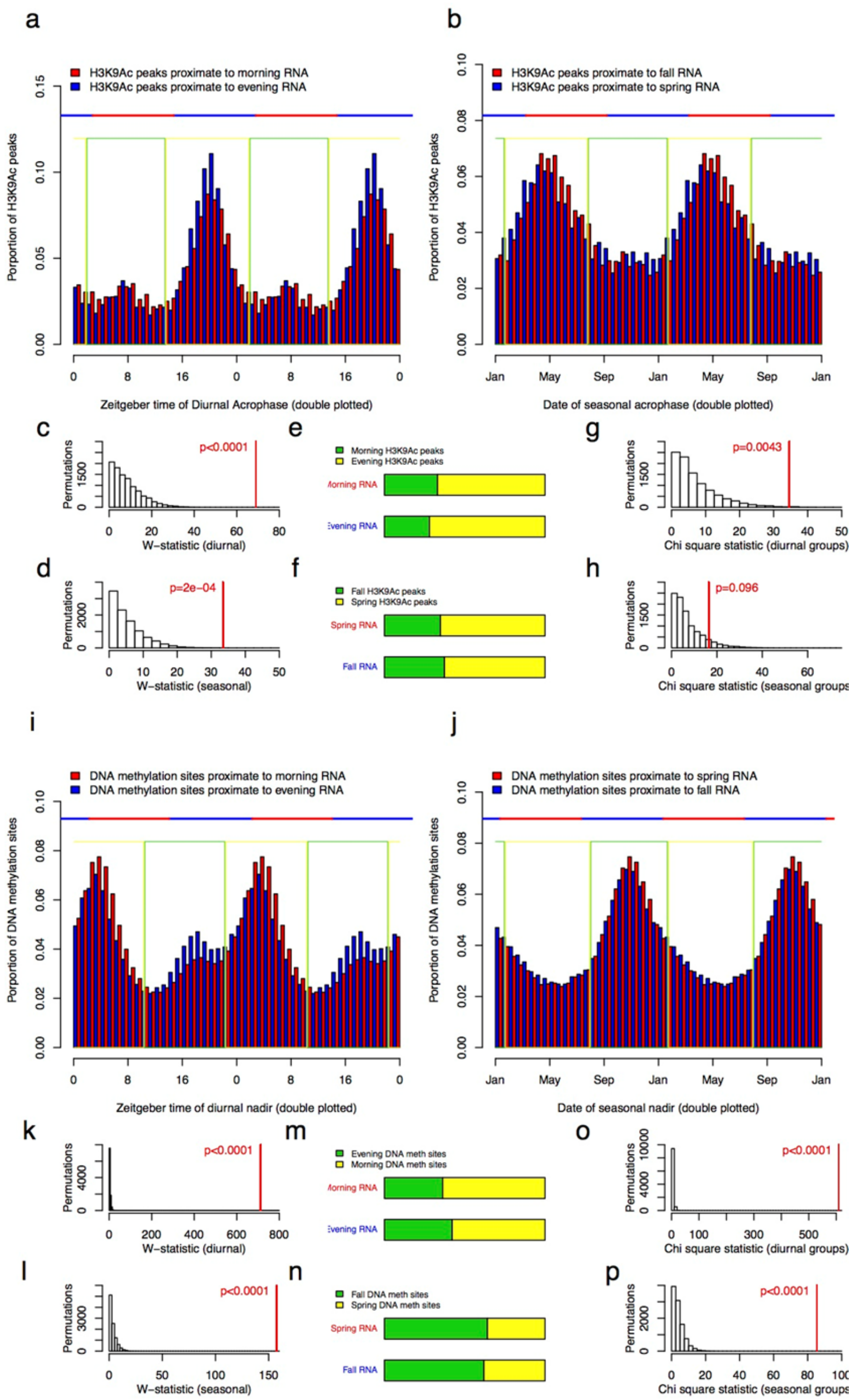

Supplementary Figure 10: Association between rhythms in the transcriptome and epigenome with diurnal rhythms referenced to local clock time. Same as Fig. 6, but reanalyzed with ZT0 = midnight, local clock time. (a): temporal distribution of H3K9Ac diurnal acrophases for peaks within 2kb of the transcription start sites of morning acrophase (red) and evening acrophase (blue) transcripts. Data are double plotted. Horizontal line indicates temporal boundaries of the associated transcript classes. Green and yellow boxes indicate temporal boundaries of H3K9Ac classes (evening yellow, morning green). (b): same as (a), but for seasonal acrophases. (c): observed vs. expected W-statistic comparing the diurnal distributions of H3K9Ac acrophases for peaks within 2kb of the transcription start sites of evening peaking and morning peaking transcripts. Expected distribution is derived from 10,000 permuted null datasets generated by randomly shuffling times of death. (d): same as (c) but for seasonal H3K9Ac acrophases. (e): diurnal temporal classification of H3K9Ac peaks near evening vs. morning peaking transcripts. (f): same as (e) for seasonal temporal classification. (g): observed vs. expected χ^2 statistic comparing the diurnal temporal classification of H3K9Ac peaks near evening vs. morning peaking transcripts. (h): same as (g) but for seasonal temporal classification. (i-p): same as (a-h) but for the diurnal and seasonal nadirs of DNA methylation sites near evening vs. morning, or fall vs. spring peaking transcripts.

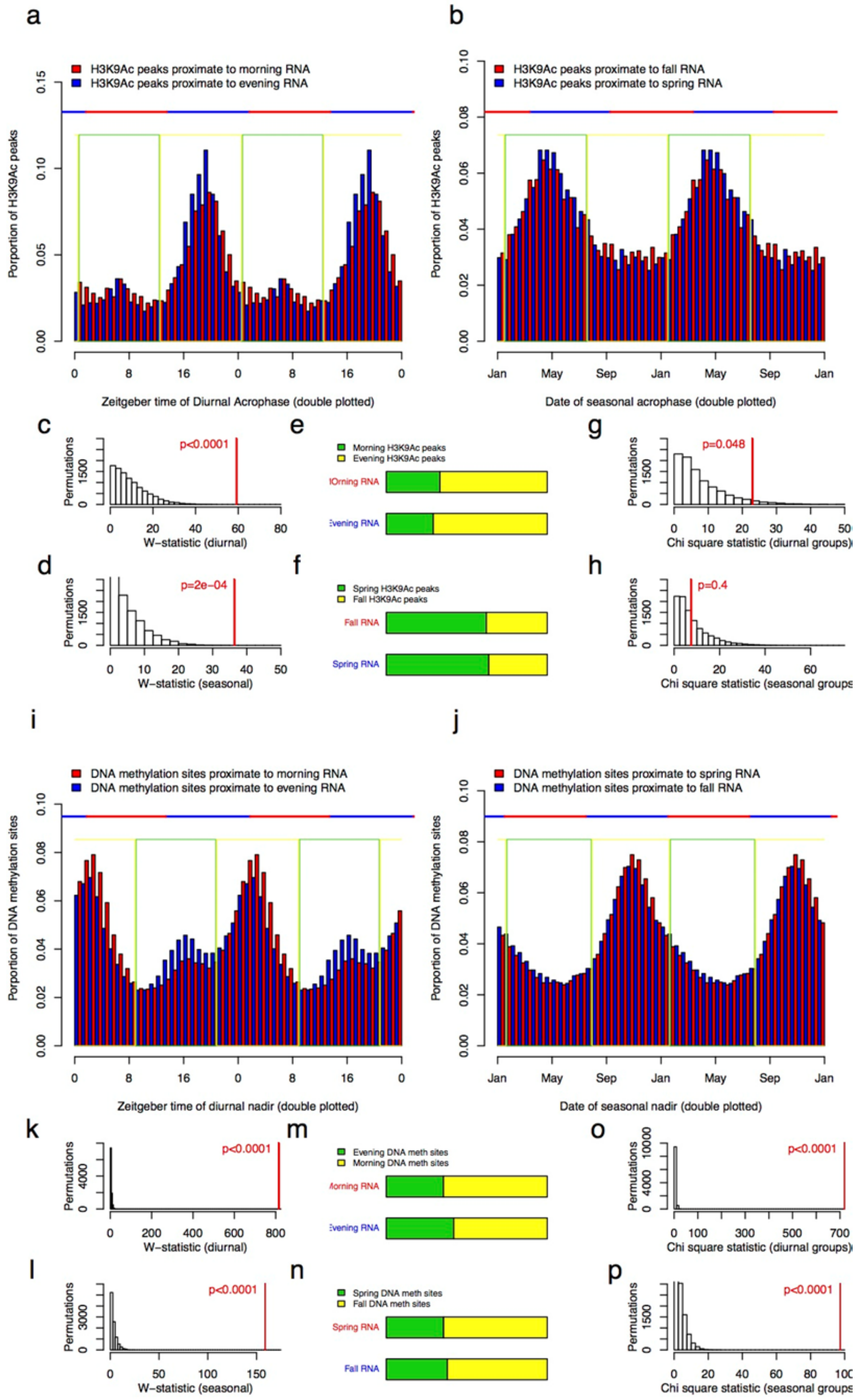

Supplementary Figure 11: Association between rhythms in the transcriptome and epigenome with diurnal rhythms referenced to the midpoint of the dark period. Same as Fig. 6, but reanalyzed with ZT0 = midpoint of dark period. (a): temporal distribution of H3K9Ac diurnal acrophases for peaks within 2kb of the transcription start sites of evening acrophase (blue) and morning acrophase (red) transcripts. Data are double plotted. Horizontal line indicates temporal boundaries of the associated transcript classes. Green and yellow boxes indicate temporal boundaries of H3K9Ac classes (evening yellow, morning green). (b): same as (a), but for seasonal acrophases. (c): observed vs. expected W-statistic comparing the diurnal distributions of H3K9Ac acrophases for peaks within 2kb of the transcription start sites of evening peaking and morning peaking transcripts. Expected distribution is derived from 10,000 permuted null datasets generated by randomly shuffling times of death. (d): same as (c) but for seasonal H3K9Ac acrophases. (e): diurnal temporal classification of H3K9Ac peaks near evening vs. morning peaking transcripts. (f): same as (e) for seasonal temporal classification. (g): observed vs. expected χ^2 statistic comparing the diurnal temporal classification of H3K9Ac peaks near evening vs. morning peaking transcripts. (h): same as (g) but for seasonal temporal classification. (i-p): same as (a-h) but for the diurnal and seasonal nadirs of DNA methylation sites near evening vs. morning, or fall vs. spring peaking transcripts.

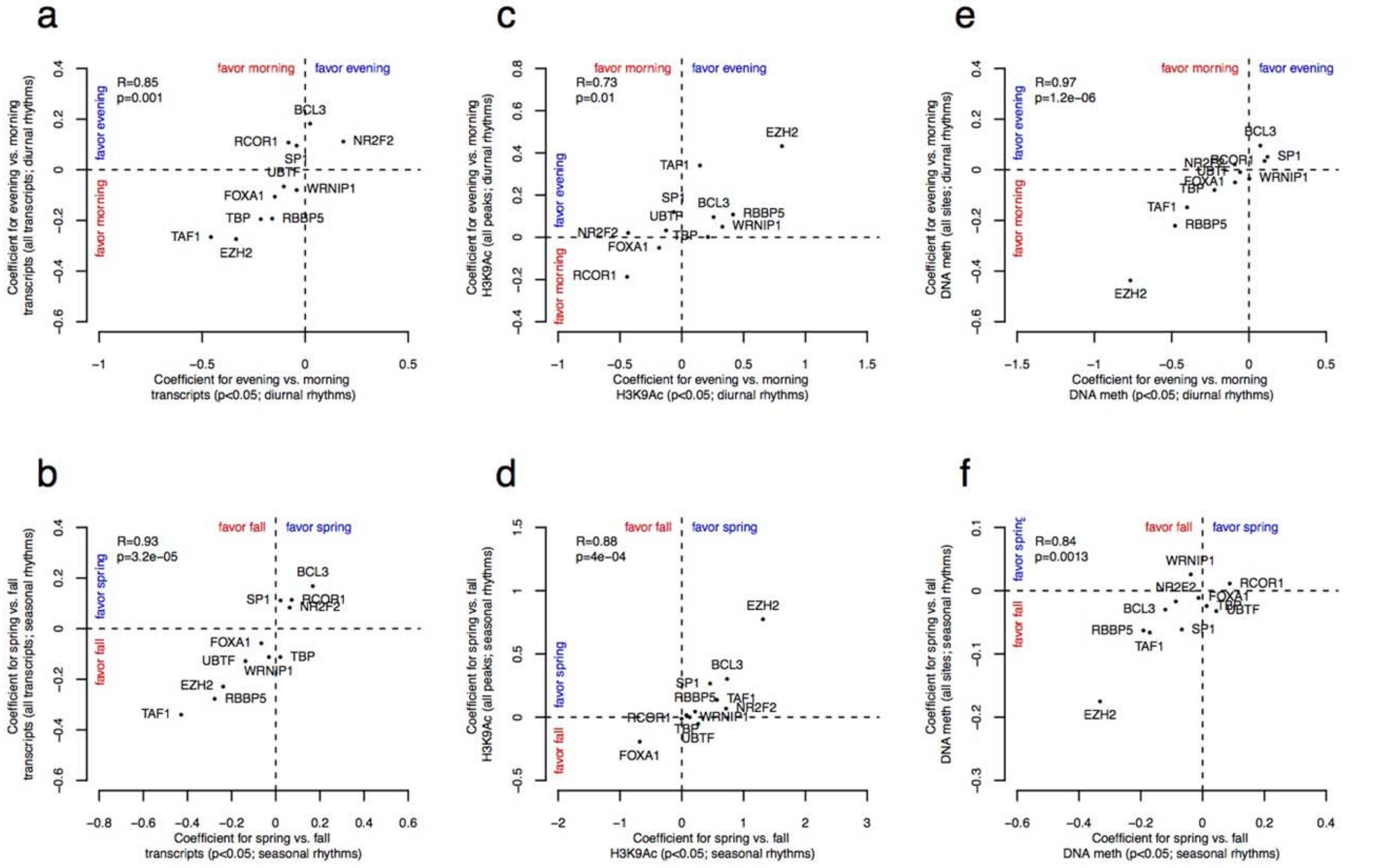

Supplementary Figure 12: Estimated effects of specific transcription factors on diurnal and seasonal based on all sites vs. only the most rhythmic sites. (a): Estimated coefficients for log(odds) of morning acrophase RNA based on all transcripts (y-axis) vs. based on only those transcripts with $p<0.05$ for diurnal rhythmicity by the F-test. (b): same as (a) but for seasonal rhythms. (c-d): as in (a-b) but for H3K9Ac peaks. (e-f): as in (a-b) but for DNA methylation sites.

a

	RNA estimated effect on log(odds) of evening vs. morning	RNA estimated effect on log(odds) of spring vs. fall	H3K9Ac estimated effect on log(odds) of evening vs. morning	H3K9Ac estimated effect on log(odds) of spring vs. fall	DNA methylation estimated effect on log(odds) of evening vs. morning	DNA methylation estimated effect on log(odds) of spring vs. fall
	Estimate (p-value) FDR	Estimate (p-value) FDR	Estimate (p-value) FDR	Estimate (p-value) FDR	Estimate (p-value) FDR	Estimate (p-value) FDR
BCL3	+2.0e-01 (<0.0001) <0.01	+1.8e-01 (<0.0001) <0.01	+1.0e-01 (0.5132) >0.99	+3.1e-01 (0.0043) 0.33	+7.9e-02 (0.1037) 0.58	-2.9e-02 (0.6035) >0.99
EZH2	-2.6e-01 (<0.0001) <0.01	-2.4e-01 (<0.0001) <0.01	+4.7e-01 (0.2964) 0.30	+7.8e-01 (0.0891) 0.09	-4.1e-01 (<0.0001) <0.01	-1.8e-01 (0.6548) >0.99
FOXA1	-9.8e-02 (0.0015) 0.04	-5.5e-02 (0.0985) 0.26	-5.0e-02 (0.6784) >0.99	-1.8e-01 (0.0491) 0.44	-4.2e-02 (0.2327) 0.66	-1.3e-02 (0.7342) >0.99
NR2F2	+9.5e-02 (0.0034) 0.08	+6.8e-02 (0.0368) 0.24	+3.5e-02 (0.5644) >0.99	+7.8e-02 (0.1762) 0.85	+1.6e-02 (0.6236) 0.83	-1.3e-02 (0.6690) 0.99
RBBP5	-2.4e-01 (<0.0001) <0.01	-2.6e-01 (<0.0001) <0.01	+1.4e-01 (0.4325) 0.93	+4.4e-02 (0.8168) 0.94	-1.9e-01 (0.2488) 0.54	-7.6e-02 (0.6483) >0.99
RCOR1	+7.1e-02 (0.0184) 0.19	+1.0e-01 (0.0005) 0.04	-1.5e-01 (0.0299) >0.99	-2.7e-02 (0.7025) 0.99	+2.3e-02 (0.5154) 0.74	+1.2e-02 (0.7312) >0.99
SP1	+1.0e-01 (0.0017) 0.07	+9.9e-02 (0.0022) 0.06	+1.5e-01 (0.3714) 0.94	+2.6e-01 (0.0605) 0.31	+4.2e-02 (0.1701) 0.63	-4.5e-02 (0.1325) >0.99
TAF1	-2.8e-01 (<0.0001) <0.01	-3.6e-01 (<0.0001) <0.01	+2.8e-01 (0.0479) 0.63	+1.3e-01 (0.4163) 0.70	-1.3e-01 (0.1415) 0.44	-6.9e-02 (0.5016) >0.99
TBP	-2.1e-01 (0.0002) <0.01	-1.4e-01 (0.0098) 0.03	-1.5e-02 (0.8416) >0.99	+3.3e-02 (0.6503) >0.99	-6.3e-02 (0.1867) 0.55	-2.9e-02 (0.5784) >0.99
UBTF	-5.7e-02 (0.0570) 0.27	-1.3e-01 (<0.0001) <0.01	+5.1e-02 (0.6924) >0.99	-4.2e-02 (0.7365) 0.94	+5.6e-04 (0.9763) >0.99	-3.0e-02 (0.0630) >0.99
WRNIP1	-8.4e-02 (0.0128) 0.09	-1.1e-01 (0.0013) 0.02	-1.5e-02 (0.8554) >0.99	-3.8e-03 (0.9597) 0.97	-4.0e-02 (0.2378) 0.63	+3.0e-02 (0.3798) >0.99

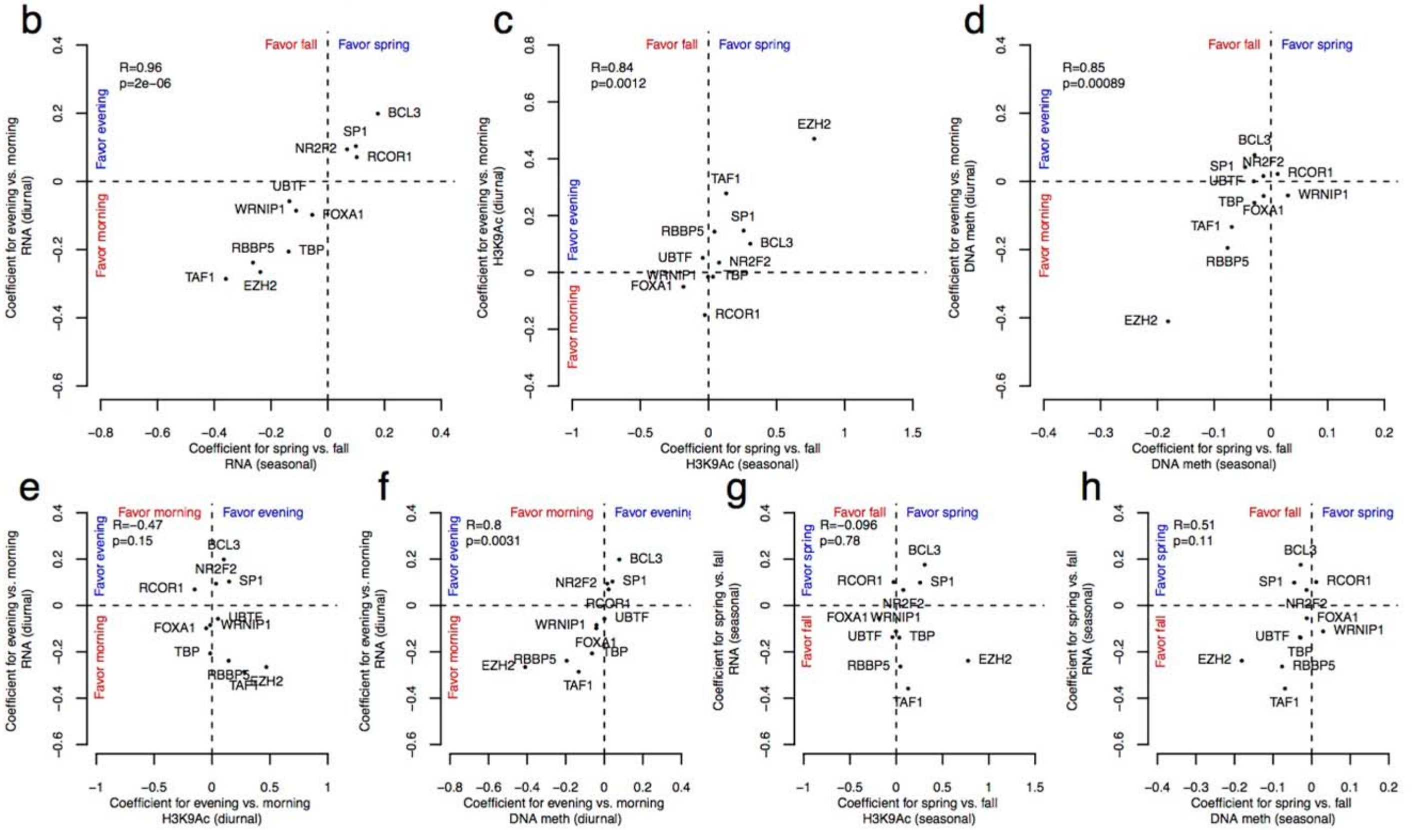

Supplementary Figure 13: Transcription factor binding sites and rhythms in the transcriptome and epigenome with diurnal rhythms referenced to local clock time. Same as Fig. 7, but reanalyzed with ZT0 = midnight, local clock time. (a): Transcription factor binding sites associated with at least one of diurnal or seasonal rhythms of RNA expression, H3K9 acetylation, or DNA methylation at FDR<0.05. Red indicates favors morning or fall timing; blue indicates favors evening or spring timing. Dark shading indicates significant at analysis FDR<0.05. Medium shading indicates significant at nominal p<0.05. Light shading indicates nominal p>0.05. (b): Estimated coefficients for log(odds) of morning acrophase RNA vs. coefficients for log(odds) of fall acrophase RNA. (c-d): same as (b) but for acrophases of H3K9Ac peaks (c) or nadirs DNA methylation sites (d). (e): estimated coefficients for log(odds) of morning acrophase RNA vs. estimated coefficients for log(odds) of morning acrophase H3K9Ac peaks. (f): same but for seasonal rhythms. (g): estimated coefficients for log(odds) of morning acrophase RNA vs. estimated coefficients for log(odds) of morning nadir DNA methylation sites. (h): same as (g) but for seasonal rhythms.

a

	RNA estimated effect on log(odds) of evening vs. morning	RNA estimated effect on log(odds) of spring vs. fall	H3K9Ac estimated effect on log(odds) of evening vs. morning	H3K9Ac estimated effect on log(odds) of spring vs. fall	DNA methylation estimated effect on log(odds) of evening vs. morning	DNA methylation estimated effect on log(odds) of spring vs. fall
	Estimate (p-value) FDR	Estimate (p-value) FDR	Estimate (p-value) FDR	Estimate (p-value) FDR	Estimate (p-value) FDR	Estimate (p-value) FDR
BCL3	+2.0e-01 (<0.0001) <0.01	+1.8e-01 (<0.0001) <0.01	+1.0e-01 (0.5132) >0.99	+3.1e-01 (0.0043) 0.33	+7.9e-02 (0.1037) 0.58	-2.9e-02 (0.6035) >0.99
EZH2	-2.6e-01 (<0.0001) <0.01	-2.4e-01 (<0.0001) <0.01	+4.7e-01 (0.2964) 0.30	+7.8e-01 (0.0891) 0.09	-4.1e-01 (<0.0001) <0.01	-1.8e-01 (0.6548) >0.99
FOXA1	-9.8e-02 (0.0015) 0.04	-5.5e-02 (0.0985) 0.26	-5.0e-02 (0.6784) >0.99	-1.8e-01 (0.0491) 0.44	-4.2e-02 (0.2327) 0.66	-1.3e-02 (0.7342) >0.99
NR2F2	+9.5e-02 (0.0034) 0.08	+6.8e-02 (0.0368) 0.24	+3.5e-02 (0.5644) >0.99	+7.8e-02 (0.1762) 0.85	+1.6e-02 (0.6236) 0.83	-1.3e-02 (0.6690) 0.99
RBBP5	-2.4e-01 (<0.0001) <0.01	-2.6e-01 (<0.0001) <0.01	+1.4e-01 (0.4325) 0.93	+4.4e-02 (0.8168) 0.94	-1.9e-01 (0.2488) 0.54	-7.6e-02 (0.6483) >0.99
RCOR1	+7.1e-02 (0.0184) 0.19	+1.0e-01 (0.0005) 0.04	-1.5e-01 (0.0299) >0.99	-2.7e-02 (0.7025) 0.99	+2.3e-02 (0.5154) 0.74	+1.2e-02 (0.7312) >0.99
SP1	+1.0e-01 (0.0017) 0.07	+9.9e-02 (0.0022) 0.06	+1.5e-01 (0.3714) 0.94	+2.6e-01 (0.0605) 0.31	+4.2e-02 (0.1701) 0.63	-4.5e-02 (0.1325) >0.99
TAF1	-2.8e-01 (<0.0001) <0.01	-3.6e-01 (<0.0001) <0.01	+2.8e-01 (0.0479) 0.63	+1.3e-01 (0.4163) 0.70	-1.3e-01 (0.1415) 0.44	-6.9e-02 (0.5016) >0.99
TBP	-2.1e-01 (0.0002) <0.01	-1.4e-01 (0.0098) 0.03	-1.5e-02 (0.8416) >0.99	+3.3e-02 (0.6503) >0.99	-6.3e-02 (0.1867) 0.55	-2.9e-02 (0.5784) >0.99
UBTF	-5.7e-02 (0.0570) 0.27	-1.3e-01 (<0.0001) <0.01	+5.1e-02 (0.6924) >0.99	-4.2e-02 (0.7365) 0.94	+5.6e-04 (0.9763) >0.99	-3.0e-02 (0.0630) >0.99
WRNIP1	-8.4e-02 (0.0128) 0.09	-1.1e-01 (0.0013) 0.02	-1.5e-02 (0.8554) >0.99	-3.8e-03 (0.9597) 0.97	-4.0e-02 (0.2378) 0.63	+3.0e-02 (0.3798) >0.99

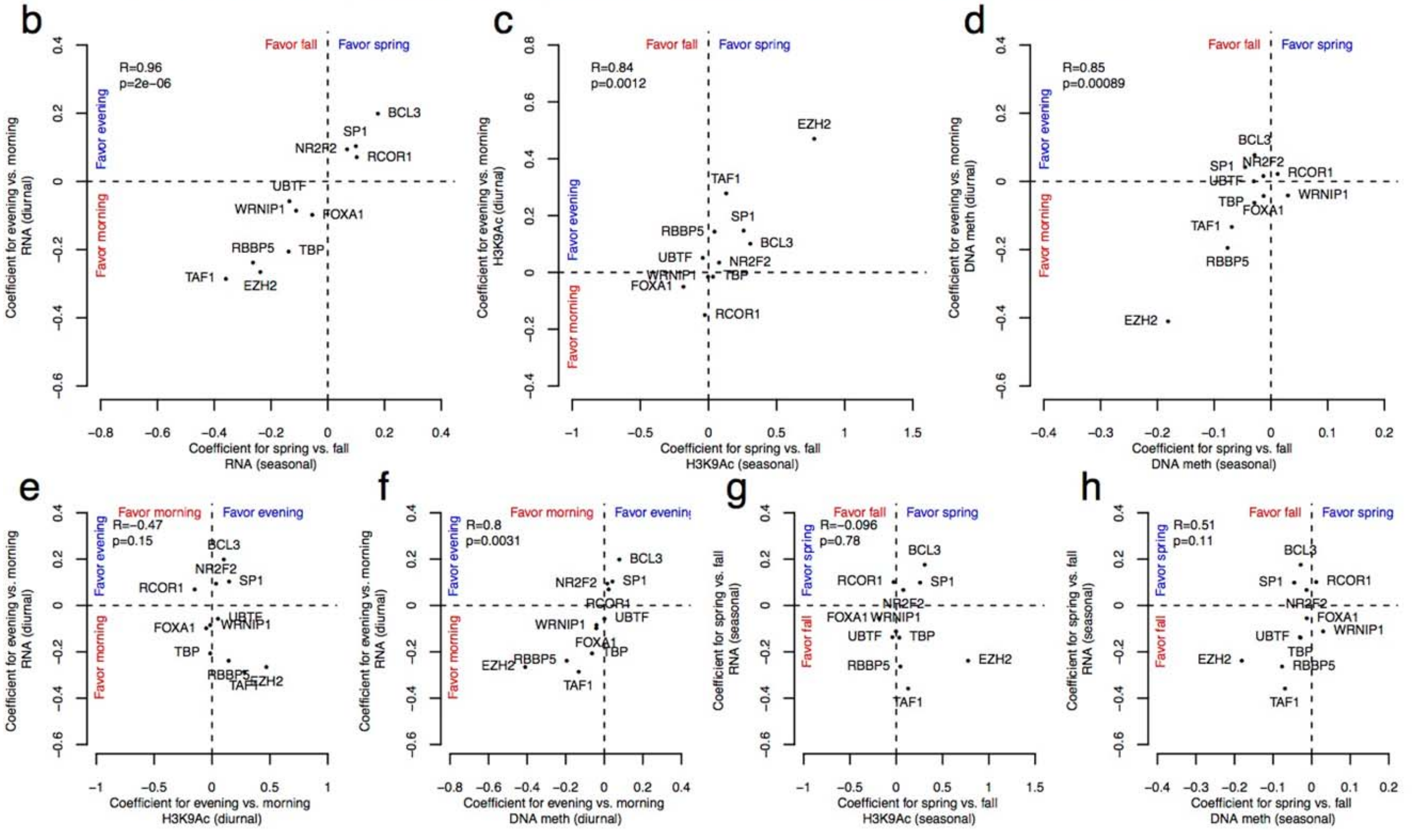

Supplementary Figure 14: Transcription factor binding sites and rhythms in the transcriptome and epigenome with diurnal rhythms referenced to the midpoint of the dark period. Same as Fig. 7, but reanalyzed with ZT0 = midpoint of the dark period. (a): Transcription factor binding sites associated with at least one of diurnal or seasonal rhythms of RNA expression, H3K9 acetylation, or DNA methylation at FDR<0.05. Red indicates favors morning or fall timing; blue indicates favors evening or spring timing. Dark shading indicates significant at analysis FDR<0.05. Medium shading indicates significant at nominal p<0.05. Light shading indicates nominal p>0.05. (b): Estimated coefficients for log(odds) of morning acrophase RNA vs. coefficients for log(odds) of fall acrophase RNA. (c-d): same as (b) but for acrophases of H3K9Ac peaks (c) or nadirs DNA methylation sites (d). (e): estimated coefficients for log(odds) of morning acrophase RNA vs. estimated coefficients for log(odds) of morning acrophase H3K9Ac peaks. (f): same but for seasonal rhythms. (g): estimated coefficients for log(odds) of morning acrophase RNA vs. estimated coefficients for log(odds) of morning nadir DNA methylation sites. (h): same as (g) but for seasonal rhythms.

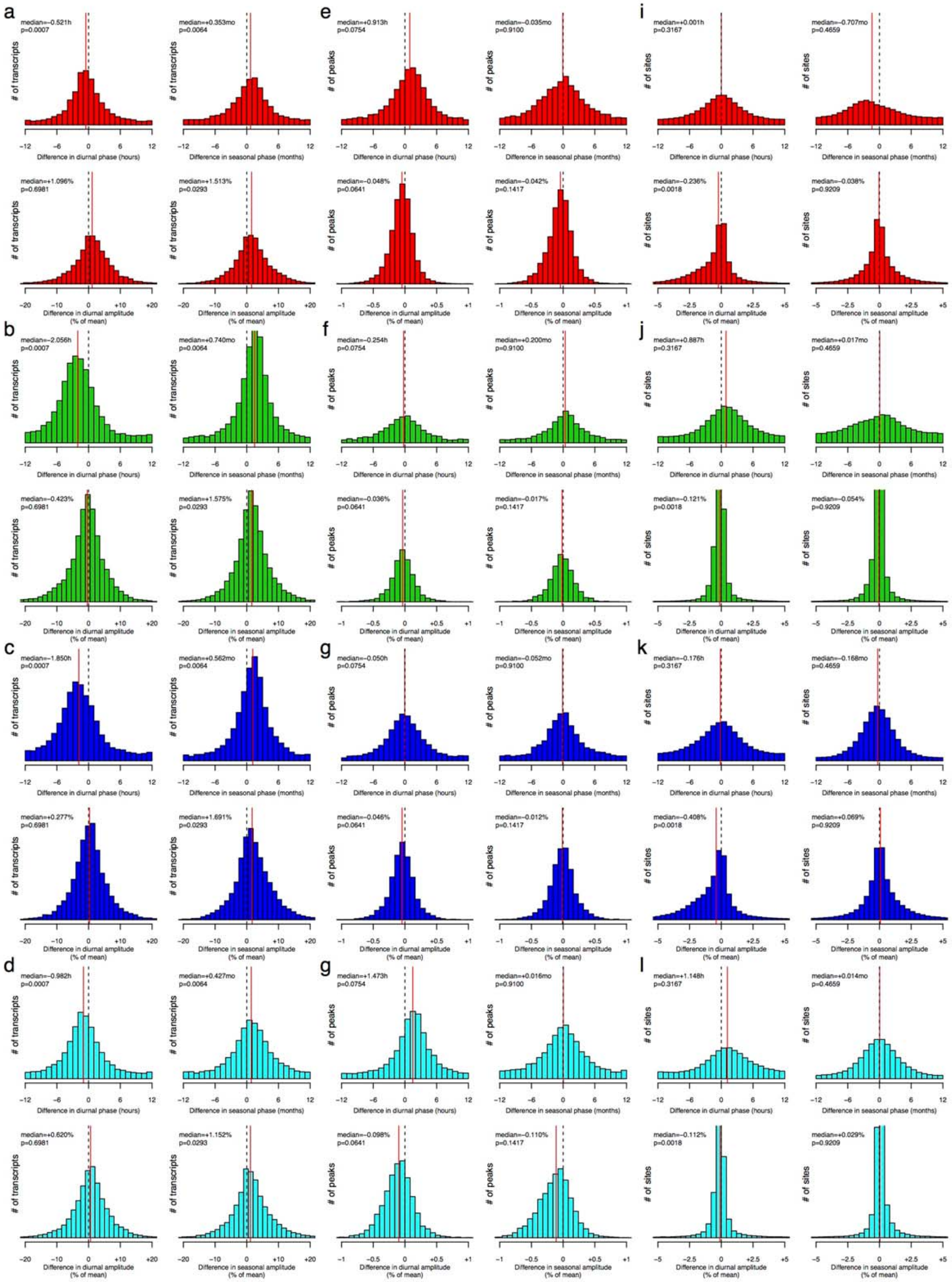

Supplementary Figure 15: Alzheimer's disease and diurnal/seasonal rhythms in the transcriptome and epigenome. Distribution of differences in timing and amplitude of transcript (a-d), H3K9Ac (e-h), and DNA methylation (i-l) rhythms for samples with Alzheimer's disease vs. samples without. Colors correspond to the groups of transcripts in Figure 8a, H3K9Ac peaks in Figure 8d, or DNA methylation sites in Figure 8g. Red lines indicate median differences. P-values calculated by comparison to 10,000 empiric null datasets generated by randomly shuffling times and dates of death.

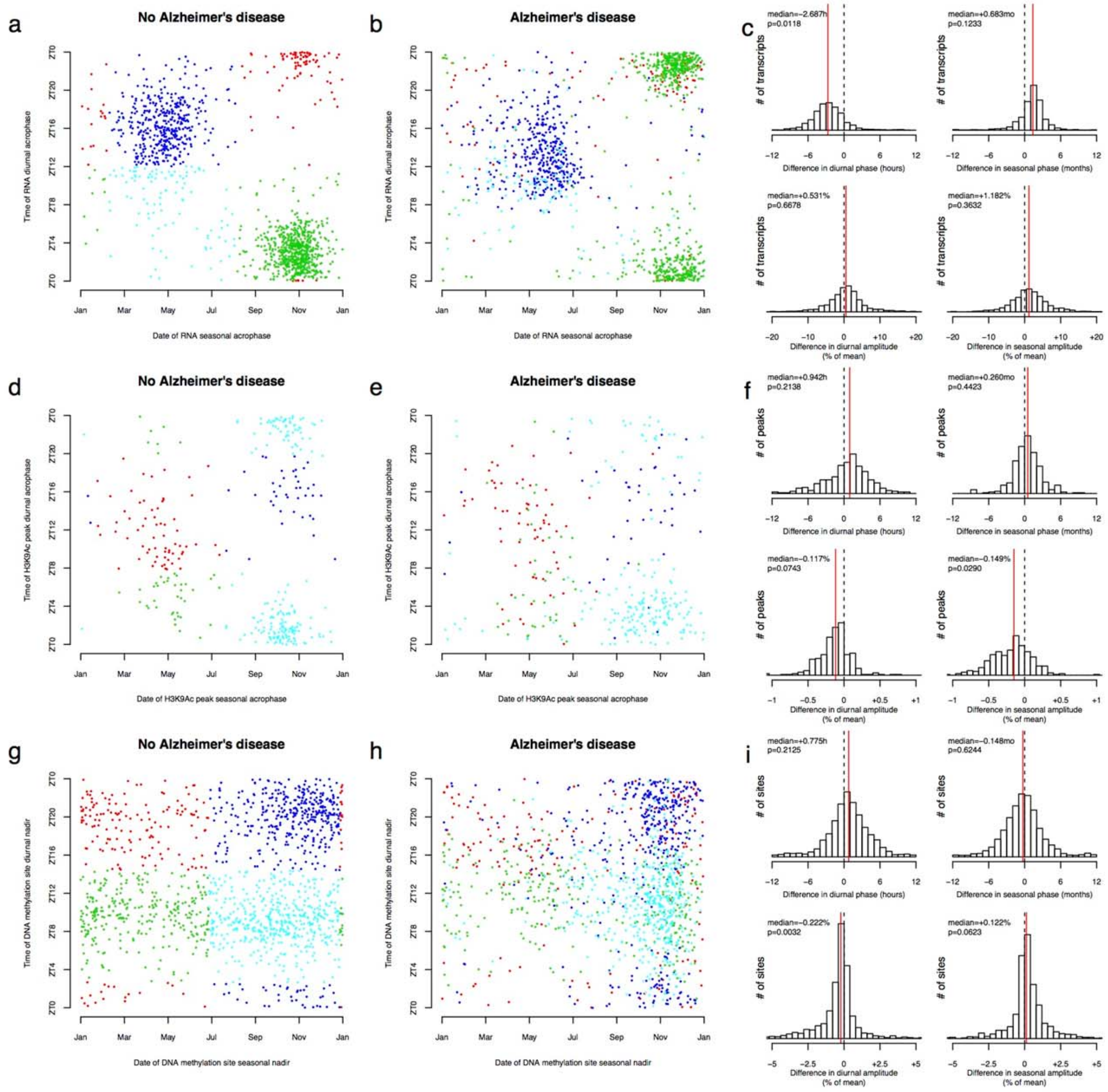

Supplementary Figure 16: Alzheimer's disease and diurnal/seasonal rhythms in the transcriptome and epigenome considering only the most rhythmic sites. Same as Fig. 8 but considering only those sites with $p<0.05$ for diurnal and seasonal rhythmicity by the F-test (a): Model-predicted times of diurnal vs. seasonal acrophases in samples without a pathological diagnosis of Alzheimer's disease. Each dot represents a single transcript. Colors depict empirically derived clusters. (b): as in (a) but for samples with a pathological diagnosis of Alzheimer's disease. Colors depict clustering based on samples without Alzheimer's disease. (c): Distribution of differences in timing and amplitude of transcript rhythms for samples with Alzheimer's disease vs. samples without. Red lines indicate median differences. P-values calculated by comparison to 10,000 empiric null datasets generated by randomly shuffling times and dates of death. (d-f): as for (a-c) but for H3K9Ac peaks. (g-i): as for (a-c) but for DNA methylation sites.

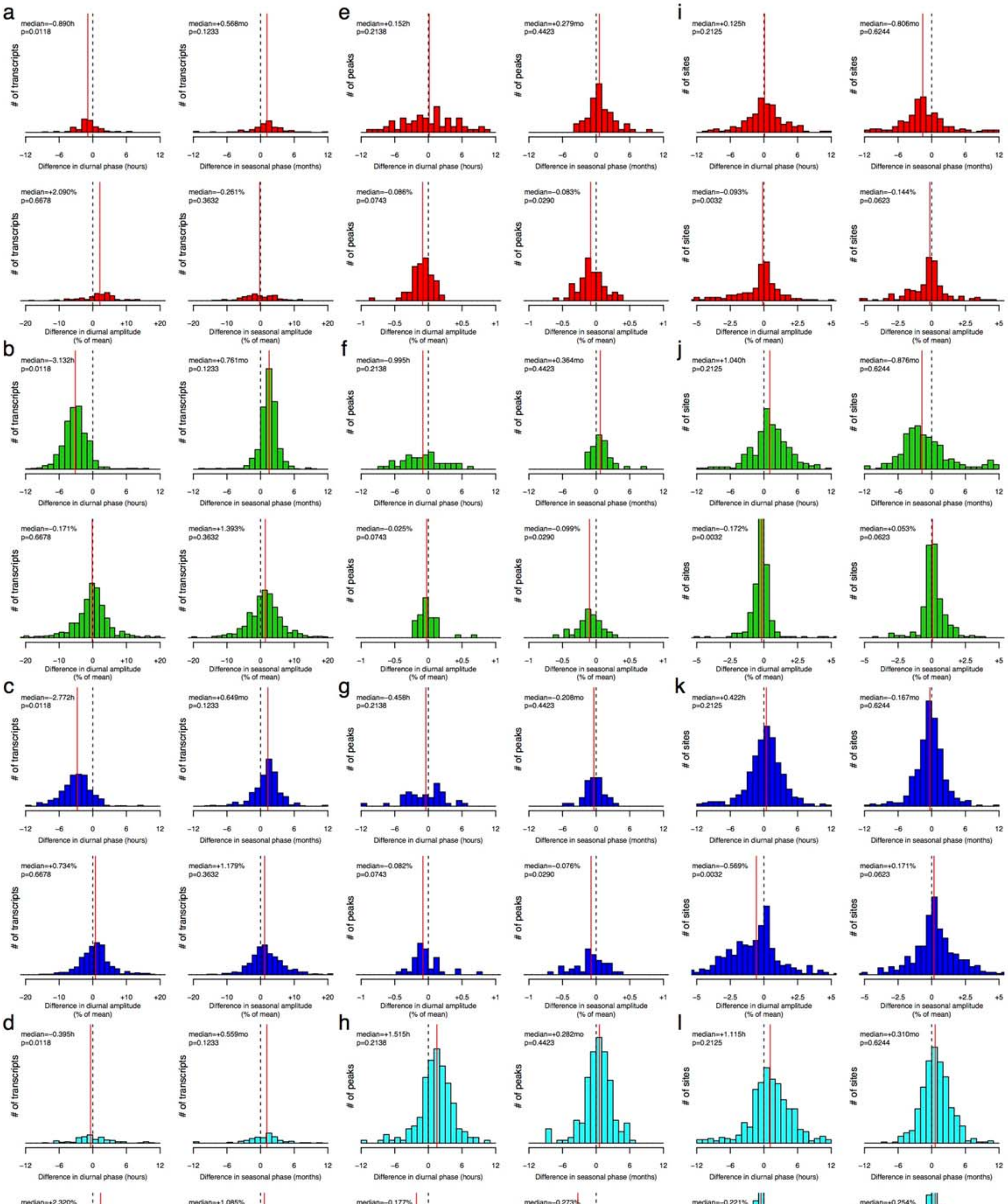

Supplementary Figure 17: Alzheimer's disease and diurnal/seasonal rhythms in the transcriptome and epigenome considering only the most rhythmic sites. Same as Supplementary Fig. 15 but considering only those sites with $p<0.05$ for diurnal and seasonal rhythmicity by the F-test**.** Distribution of differences in timing and amplitude of transcript (a-d), H3K9Ac (e-h), and DNA methylation (i-l) rhythms for samples with Alzheimer's disease vs. samples without. Colors correspond to the groups of transcripts in Supplementary Fig. 16a, H3K9Ac peaks in Supplementary Fig. 16d, or DNA methylation sites in Supplementary Fig. 16g. Red lines indicate median differences. P-values calculated by comparison to 10,000 empiric null datasets generated by randomly shuffling times and dates of death.

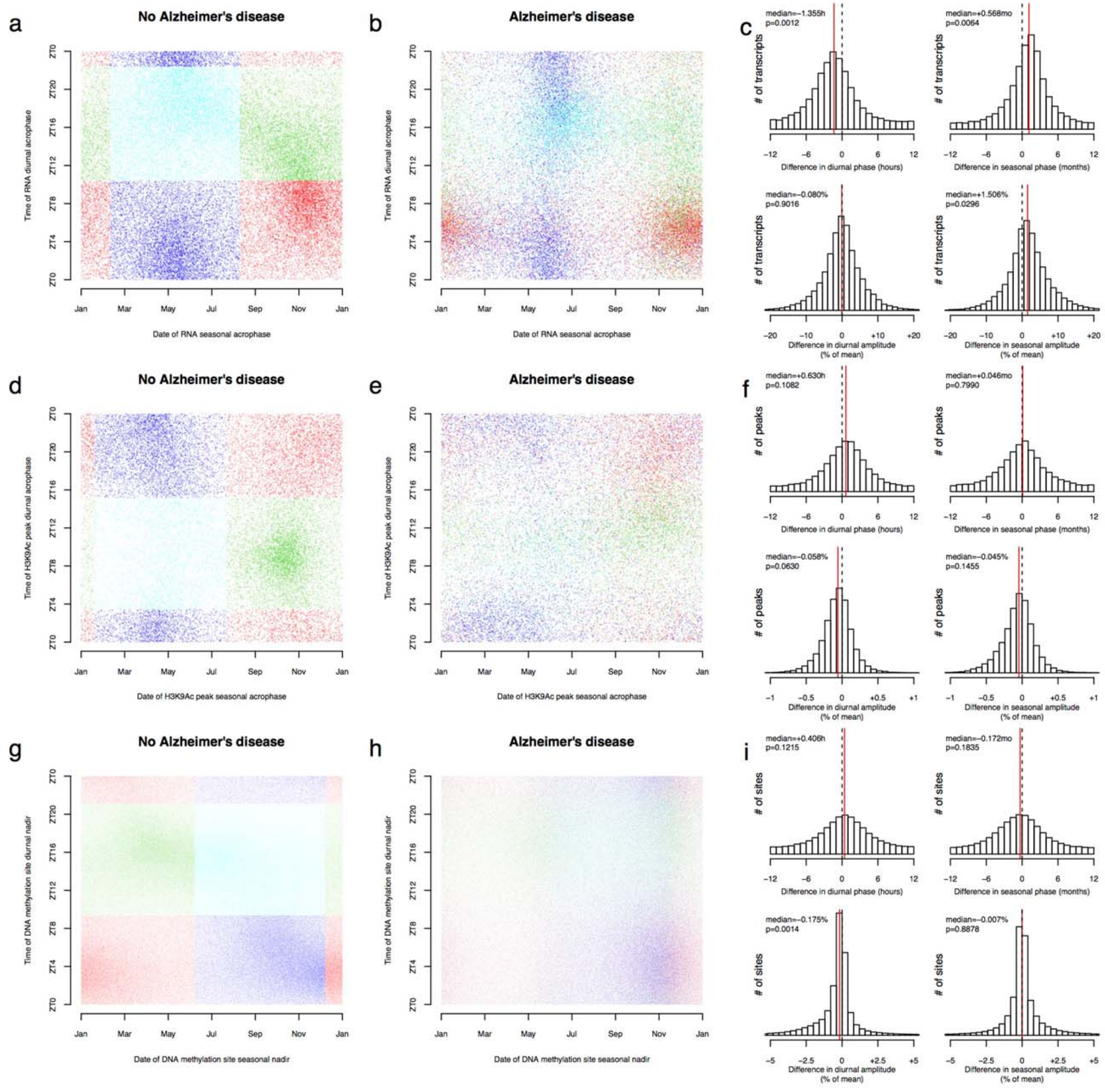

Supplementary Figure 18: Alzheimer's disease and diurnal/seasonal rhythms in the transcriptome and epigenome with diurnal rhythms referenced to local clock time. Same as Fig. 8, but reanalyzed with ZT0 = midnight, local clock time. (a): Model-predicted times of diurnal vs. seasonal acrophases in samples without a pathological diagnosis of Alzheimer's disease. Each dot represents a single transcript. Colors depict empirically derived clusters. (b): as in (a) but for samples with a pathological diagnosis of Alzheimer's disease. Colors depict clustering based on samples without Alzheimer's disease. (c): Distribution of differences in timing and amplitude of transcript rhythms for samples with Alzheimer's disease vs. samples without. Red lines indicate median differences. P-values calculated by comparison to 10,000 empiric null datasets generated by randomly shuffling times and dates of death. (d-f): as for (a-c) but for H3K9Ac peaks. (g-i): as for (a-c) but for DNA methylation sites.

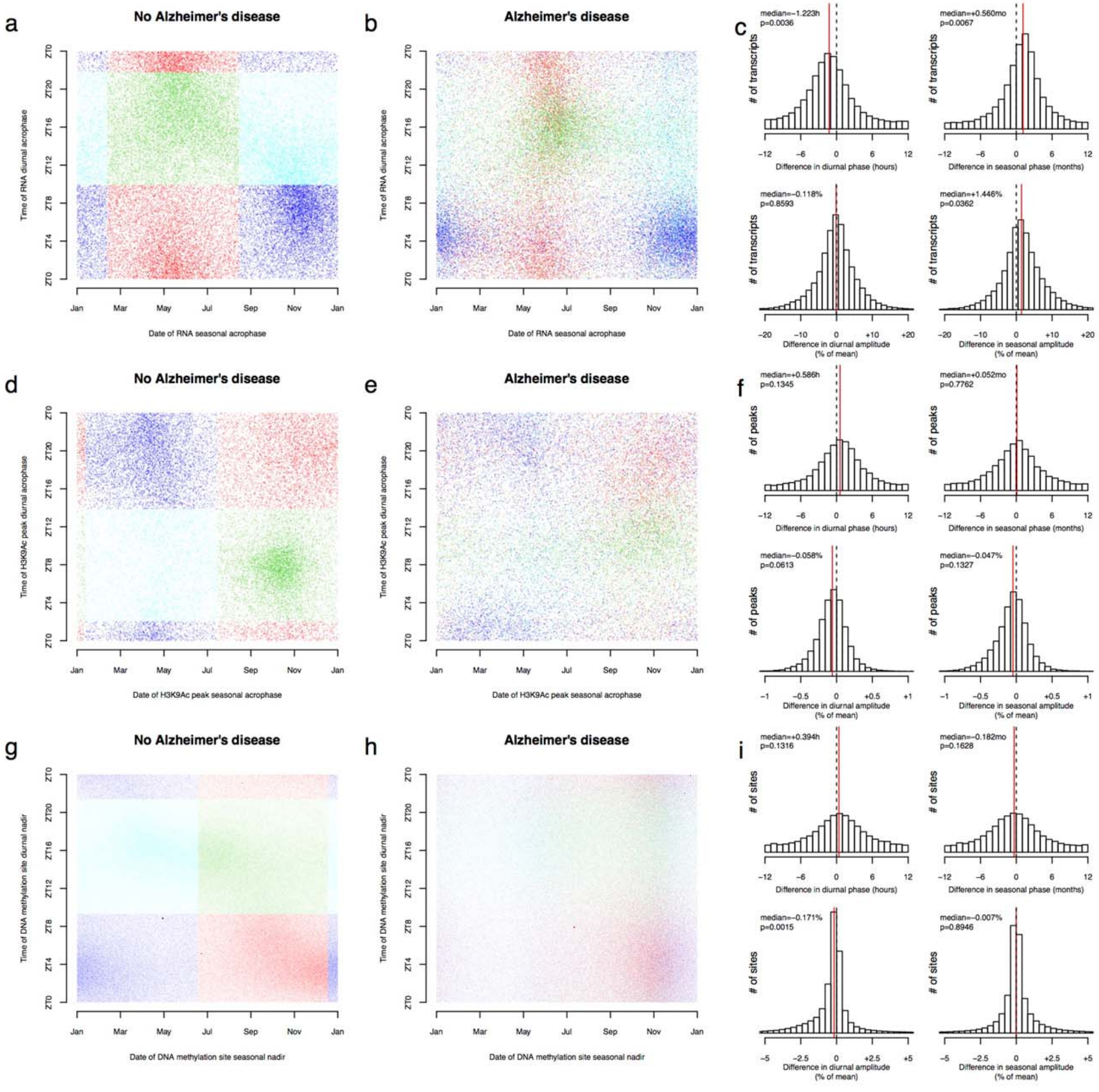

Supplementary Figure 19: Alzheimer's disease and diurnal/seasonal rhythms in the transcriptome and epigenome with diurnal rhythms referenced to the midpoint of the dark period. Same as Fig. 8, but reanalyzed with ZT0 = midpoint of the dark period. (a): Model-predicted times of diurnal vs. seasonal acrophases in samples without a pathological diagnosis of Alzheimer's disease. Each dot represents a single transcript. Colors depict empirically derived clusters. (b): as in (a) but for samples with a pathological diagnosis of Alzheimer's disease. Colors depict clustering based on samples without Alzheimer's disease. (c): Distribution of differences in timing and amplitude of transcript rhythms for samples with Alzheimer's disease vs. samples without. Red lines indicate median differences. P-values calculated by comparison to 10,000 empiric null datasets generated by randomly shuffling times and dates of death. (d-f): as for (a-c) but for H3K9Ac peaks. (g-i): as for (a-c) but for DNA methylation sites.
